# News

**Published:** 2011-12

## News

### Sad news

**Figure:**
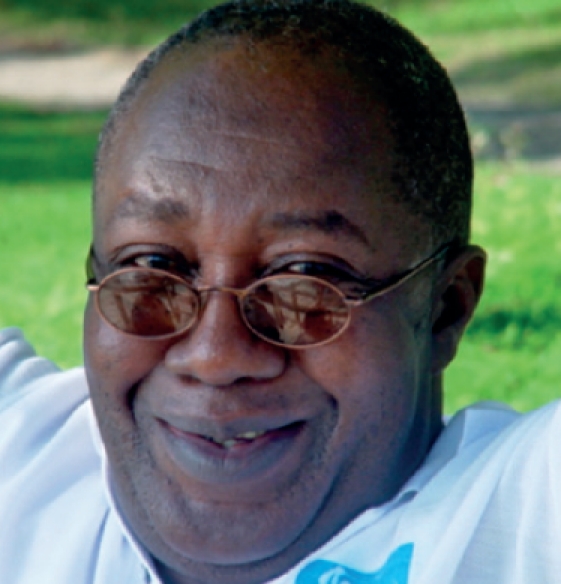


The much-admired ophthalmologist Dr Dennis Williams died earlier this year. Dr Williams was born in Nigeria but educated in Sierra Leone, and was the only ophthalmologist to remain in the country during the war. He worked for Sightsavers for much of his life, and was appointed Vice President of Sightsavers on his retirement in 2008; the only staff member ever to receive this honour. He will be remembered for this courage, his leadership, his humour, and his humility. Dr Williams, who was suffering from cancer, is survived by his wife, Pamela, and four children.

### Have your say

Our September 2012 issue is about glaucoma and visual fields. Have you had a useful or interesting experience you would like to share with other readers? Do you have any questions you would like to ask our experts? Write to: The Editor, International Centre for Eye Health, London School of Hygiene and Tropical Medicine, London WC1E 7HT, UK. Email: editor@cehjournal.org Deadline: 15 March 2012.

### Community Eye Health Update CD

**Figure:**
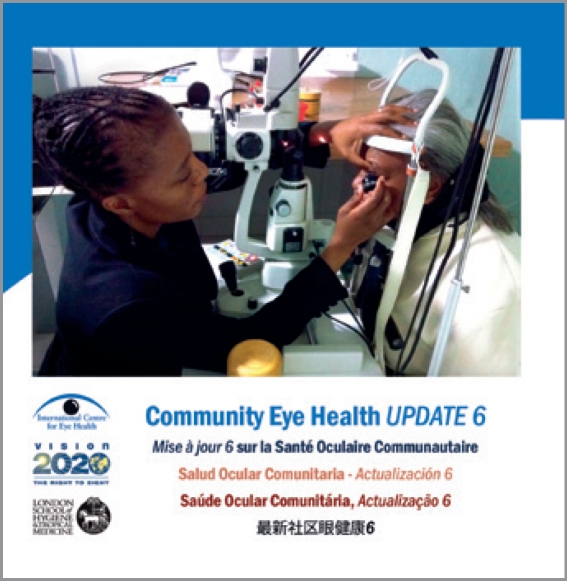


Look out for your free copy of the Community Eye Health Update CD in this issue of the journal. It contains back issues of the journal in English, French, Spanish, Portuguese, and Chinese, as well as photographs, books, software, and many other helpful resources. If you have not received your copy, please contact TALC (see page 30) to request a replacement.

### Get your own copy

Do you get your own copy of the *Community Eye Health Journal?* Do you know anyone else who would like their own, free copy? Or have you moved or changed jobs? Send your up-to-date details to Anita Shah, International Centre for Eye Health, London School of Hygiene and Tropical Medicine, London WC1E 7HT, UK.

Email: admin@cehjournal.org

## Courses

### Community Eye Health Institute, University of Cape Town, South Africa

For information about VISION 2020 certificate courses in 2012, a postgraduate diploma in community eye health (PGDip) in 2013, or a Masters in Public Health (community eye health) in 2013, contact Zanele Magwa, Community Eye Health Institute, University of Cape Town, Private Bag 3, Rondebosch 7700, South Africa. Tel: +27 21 404 7735. Email: ntombizanele.magwa@uct.ac.za

### International Centre for Eye Health

**MSc in Public Health for Eye Care.**

From September 2012 to September 2013 or part-time over two years. Apply before April 2012. For scholarships and details of application, write to: Registry, LSTHM, Keppel Street, London WC1E 7HT, UK. Tel: +44 207 299 4646 or visit **www.lshtm.ac.uk/prospectus/masters/mscphec.html**

**New short course: Understanding an eye health system in order to achieve VISION 2020.** A five-day course to familiarise participants with a health systems approach to eye care in developing countries, through using practical interactive examples and case studies. Start date: June 25th 2012. Duration: 5 days. Cost: £915. Place: London. This course is ideal for eye care professionals who wish to work or are working in low- and middle-income countries.

### Kilimanjaro Centre for Community Ophthalmology (KCCO), Tanzania

For information on courses, contact Genes Mng'anya, KCCO, Good Samaritan Foundation, PO Box 2254 Moshi, Tanzania. Tel: +255 27 275 3547. Email: genes@kcco.net or visit www.kcco.net

### Lions SightFirst Eye Hospital, Nairobi, Kenya

**Small incision cataract surgery for ophthalmologists wishing to upgrade from ECCE.** Duration: 1 month. Courses run every month. Cost: US $1,000 for tuition and US $500–700 for accommodation and meals. Write to: The Training Co-ordinator, Lions Medical Training Centre, Lions SightFirst Eye Hospital, PO Box 66576-00800, Nairobi, Kenya, call +254 20 418 32 39, or email training@lionsloresho.org

### Lions Aravind Institute of Community Ophthalmology

**Instrument maintenance courses** with a trainee: trainer ratio of 1:1. Courses start on 1 Feb, 1 Apr, 1 June, 1 Aug, 1 Oct and 1 Dec 2012. Duration: Four weeks. Cost: US $400 (including tools). Visit **www.aravind.org/education/coursedetails.asp** or write to: Prof V Srinivasan, LAICO, 72, Kuruvikaran Salai, Gandhi Nagar, Madurai 625 020, Tamil Nadu, India. Email: v.srinivasan@aravind.org

